# Level playing field: young males, masculinity and mental wellbeing through sport

**DOI:** 10.1186/s12889-022-13200-1

**Published:** 2022-04-14

**Authors:** Murray Drummond, Ben Wadham, Ivanka Prichard, Sam Elliott, Claire Drummond, Sarah Crossman

**Affiliations:** 1 College of Education, Psychology and Social Work, SHAPE Research Centre, Flinders University, Bedford Park, South Australia Australia; 2 College of Education, Psychology and Social Work, Flinders University, SA Bedford Park, Australia

## Abstract

In Australia, and throughout the world, it is evident that the mental health and wellbeing of young males aged 15–24, is not a priority. In Australia suicide is the leading cause of death in people aged 15–24 years and 75% are male (Australian Institute of Health and Welfare. Australian hospital statistics 2011–12, 2021). It is clear young males as well as those who identify as indigenous or LGBTIQ are at risk groups with respect to self arm and suicide (Drummond, MJN, et al. 2019). It is the transition period from adolescence to adluthood that is of particular concern. Often young males must pass through this phase of life with minimal guidance or direction and without a “safe space” where they can attain emotional support. Sport is a significant part of boys’ and young males' lives and offers that “safe space”. Sport can play a substantial role in protective mental health through socialization and engagement in a socially endorsed activity that seemingly has far more positive outcomes than negative ones. This research is underpinned by both quantitative and qualitative research with young males involved masculinised sporting clubs. Its aim is to provide insights into how we can create safe spaces for males and influence positive forms of masculinities that can enhance mental health promotion among young males. This mixed methods research explores issues around mental health in young males involved across two male dominated sporting codes in Australian (Australian football and cricket). Surveys and interviews with young males age 15–24 as well as parents, coaches and key stakeholders underpin this research. While the data is designed to assist in the development of educational resources for males to influence positive forms of masculinities that can enhance mental health promotion among young males involved in these sporting codes, this paper reports on the exploratory nature of the data and raises important issues emerging among young males with respect to mental health and the role of the sporting club.

## Background

The mental health and wellbeing of young males aged 15–24, globally, is not being adequately addressed. The Australian Bureau of Statistics reports that intentional self-harm is the leading cause of death among Australian children and young people aged 15–24 years, with 75% being male [1]. Young males have a significant risk of self-harm and suicide, while those who identify as Indigenous and LGBTIQ are at even greater risk [2]. Unlike the transition period from childhood to adolescence, where boys are relatively insulated within the constructs of their school and family environment [3], the transition phase through adolescence to early adulthood is less controlled and requires immediate attention. Many young males must navigate this part of their lives alone without ‘safe spaces’ where they can attain support for various physical and emotional needs [3].

Sport is a significant part of boys’ and young males' lives and offers that safe space. Sport can play a substantial role in protective mental health through socialization and engagement in a socially endorsed activity that seemingly has far more positive outcomes than negative ones[3]. However, while sport is viewed favourably as a site for the social construction of masculinity in young males [3–6], there are aspects of certain sports that are recognized as traditionally hegemonic masculine environments, which champion characteristics such as heterosexuality, muscularity, and non-feminist ideologies (e.g. prize money for men should be more) to name a few [7–11]. These are particularly so for Australian football, rugby, and cricket and other historically male dominated sports where fraternal bonding is traditionally strong [12, 13]. Consequently, these socially constructed masculinised spaces can establish, endorse, and perpetuate hegemonic masculine ideals (e.g. strength, power, dominance, stoicism) that may ultimately provide barriers to a more open and fluid masculine ideology [3]. Given that mental health concerns are often perceived as feminised conditions [14], reframing the meaning of masculinities through fraternal settings such as masculinised sporting environments offers a significant opportunity to redress traditional notions of masculinity and mental wellbeing within these masculinised spaces.

Sport is seen as a rite of passage for many young males [3]. Engaging boys and young males in sport and keeping them involved in activity is crucial to physical health [3]. However, having them remain engaged in a nurturing and socially responsible environment is key to ongoing mental health and wellbeing. Sporting clubs, particularly masculinised sports, have tended to espouse traditional hegemonic masculinised ideologies, which do not promote tolerance, inclusivity [12] and mental health and wellbeing. By changing these traditional hegemonic masculinised environments, these sporting clubs could potentially have the opportunity to offer spaces for young men to thrive emotionally within inclusive and understanding environments.

Young males are also described as developing limited mental health capacities, particularly concerning self-awareness, interpersonal relationship, self-regard, and empathy [15]. While sporting cultures are traditionally strong in supporting the growth of young men’s physical and social capacity [3], we need to explore how versions of manhood contribute to young males’ mental health. The research that underpins this paper focuses on the mental health literacy of young men to mental health. Competencies surrounding mental health and wellbeing are the foundation to how young men understand their relationships with themselves and others [16]. People with limited mental health literacy can fail to disclose or share mental health and vulnerability to friends, peers, and family. However, allowing young males to explore their mental health and wellbeing with a view to developing positive mental health needs to occur through multi-layers and levels. Indeed, the basis of this paper suggests that sport can provide a unique site in which this can take place. Further, the data that has been collected will provide the information to build the “tools” for this to occur.

### Aims

The underpinning aim of this study was to explore the views of young males and key stakeholders within sporting club settings to better understand young males’ mental health and wellbeing needs.

Specifically, the research aimed:

- To listen to the voices of young males around mental health and wellbeing within masculinised sporting cultures
- To explore how sporting clubs currently work with young males around mental health and wellbeing
- To investigate how masculinities are created, maintained, and perpetuated within masculinised sporting clubs

## Method

The study used a mixed-method approach, comprising two phases. *Phase One* involved a quantitative online survey that explored the perceived issues faced by young males in contemporary society across two sports and examined potential differences across these sports. *Phase Two* used qualitative interviews to gain a deeper understanding of the issues presented in Phase One.

### Phase One

#### Procedure

Phase One of the study used an online questionnaire (approximately 15–20 min) within two South Australian sporting clubs; one cricket club and one Australian Rules Football club tailored to young people (15–24 years) who participate in club sport. The questionnaire included items on mood in the last month, how much support was received from their family and other members of their sporting club, attitudes toward mental health issues in general and their perception of societal norms and roles of men and women. The sample size was based on the number of athletes available in the two clubs. Given the small pool of potential participants, no specific hypotheses were made, and the results are described descriptively.

Following ethics approval from the University’s Human Research Ethics Committee, the club administration staff distributed the survey to their membership using an email script that included a description of the project, its aims, the information sheet and a link to the survey. To complement the email recruitment method, some researchers also attended some of the club’s training sessions with an iPad to encourage survey participation. Participation in this study was entirely voluntary. The participants were required to indicate their consent to be involved, and where the participant was under 18 years of age, consent from a parent or guardian was required. Participants were given the opportunity to withdraw at any time.

### Measures

*Club and Family Support.* To measure a young person’s perception of support provided by their sporting club and family members, we used a 5-item Club Support scale, and a 4-item Family Support scale developed for the present study. Both scales were based on the Multidimensional Scale of Perceived Social Support (MSPSS) developed by Zimet, Dahlem [17]. The wording of the items was adapted to focus the respondent on support provided by either their sporting club or their family (e.g., “I feel a sense of comradeship (or closeness) between myself and other people at my club” and “I can talk about my problems with my family" respectively). The items were presented on a 5-point Likert-type scale ranging from *Strongly disagree (1)* to *Strongly agree (5)*. Responses were summed and divided by 5 (Club Support) and 4 (Family Support) to obtain a total mean score of perceived support for each type of support. Higher scores indicated higher levels of perceived support. According to Zimet, Dahlem [17], the Multidimensional Scale of Perceived Social Support has good internal consistency, with a Cronbach alpha coefficient of 0.88. In this study, the Cronbach alpha coefficient was 0.87 for both the Club Support and the Family Support scales.

*Male Role Norms.* This study used a 12-item scale to deduce attitudes towards men’s expected behaviour, based on the Male Role Norms Inventory – Short Form (MRNI-SF) [18]. The items were presented on 7-point Likert-type scales ranging from *Strongly disagree (1)* to *Strongly agree (7)*, and responses were averaged to get a total mean score for views on expected male behaviour. An example item is “A man should never admit when others hurt his feelings.” Higher scores indicated a more traditional view of men’s roles in society. The MRNI-SF was reported to have good internal consistency, with a Cronbach alpha coefficient of 0.96 [18]. In this study, the Cronbach alpha coefficient was 0.82.

*Attitudes Towards Women.* This study used the 12-item Attitudes Towards Women Scale for Adolescents (AWSA) developed by Galambos, Petersen [19]. The items (e.g., Boys are better leaders than girls”) were presented on 4-point Likert-type scales ranging from *Strongly agree (1)* to *Strongly disagree (4)*, with relevant items reverse scored. The scores were averaged to get a total mean score for attitudes toward women. Higher scores indicated an egalitarian view, while lower scores indicated a more traditional view of women’s roles in society. Galambos, Petersen [19] reported high internal consistency across three samples of adolescents, with Cronbach alpha coefficients ranging from 0.62 to 0.86. In this study, the Cronbach alpha coefficient was 0.80.

*Psychological Distress.* The 10-item Kessler Psychological Distress Scale (K10) [20] was used to identify levels of respondent’s psychological distress. Participants were asked how often they had felt certain ways in the last 30 days on 5-point Likert-type scales ranging from *None of the time (1)* to *All of the time (5)*. For example, how “nervous’ or “depressed” they had felt. The scores were averaged to get a total mean score of psychological distress. Higher scores indicated significant levels of psychological distress, while lower scores indicate lower levels of psychological distress. According to Kessler, Andrews [20], the K10 scale has good internal consistency with a Cronbach alpha coefficient of 0.93. In this study, the Cronbach alpha coefficient was 0.91.

*Mental Health Literacy Confidence.* The 4 confidence items from the 35-item Mental Health Literacy (MHL) scale, developed by O'Connor and Casey [21] were used to assess participants’ capacity to access mental health information. The items (e.g., “I am confident that I know where to seek information about mental illness”) were presented on 5-point Likert-type scales ranging from *Strongly disagree (1)* to *Strongly agree (5)*. The scores were averaged to get a total mean score for Mental Health Literacy confidence, where higher scores indicated greater confidence. O'Connor and Casey [21] reported the 35-item MHL scale has good internal consistency with a Cronbach alpha coefficient of 0.87. In this study, the Cronbach alpha coefficient of the 4 confidence items was 0.86.

*Help seeking Self-Stigma.* Vogel, Wade, and Haake’s [22] Self-Stigma of Seeking Help 10-item scale was used to measure levels of self-stigma around seeking psychological help. The items were presented on 5-point Likert-type scales ranging from *Strongly disagree (1)* to *Strongly agree (5)*, with relevant items reverse scored (e.g., “I would feel okay about myself if I made the choice to seek professional help”). The scores were averaged to get a total mean score, where higher scores indicate greater Self-Sigma. Studies to test the reliability of the Self-Stigma of Seeking Help scale reported good internal consistency, with the Cronbach alpha coefficient between 0.86 and 0.91 [22]. In this study, the Cronbach alpha coefficient was 0.83.

### Data analysis

IBM SPSS Statistics software package was used to analyse the survey data. Individual sample T-tests were used to discern differences between cricket and football participants in the established scales and subscales.

### Results: Phase One

#### Characteristics of the sample

The final dataset included 34 male participants aged 15–24 years (*M* = 18.91 years, *SD* = 2.86). The main sport of 15 participants was cricket, while 19 played Australian Rules Football (AFL). Most of the young men were Caucasian (*N* = 32), one participant was Asian, one was Indian, and almost all identified as heterosexual (*N* = 32).

Table 1 shows the means (and standard deviations) across all variables measures for the whole sample as well as by sporting type. The results were split by sporting type to provide more detailed information about the sample, however, no specific predictions were tested regarding sport type due to the small sample size. Overall, young male participants perceive both sporting clubs and their family provide them with high levels of support, with family support rated slightly higher. The top-rated item in relation to perceived club support for both cricket and football participants was feeling a sense of comradeship (*M* = 4.26, *SD* = 0.71). In relation to family support, participants felt that their families tried to help them overall (*M* = 4.71, *SD* = 0.52) and were willing to help them make decisions (*M* = 4.68, *SD* = 0.53).

**Table 1:** Total means (and standard deviations) for young male participants across all scales by the primary sport played

	**Whole sample** **(*N* = 34)**	**Cricket** **(*N* = 15)**	**Football** **(*N* = 19)**
Club Support ^a^	3.96 (0.63)	3.70 (0.70)	4.18 (0.48)
Family Support ^a^	4.58 (0.55)	4.57 (0.57)	4.59 (0.54)
Male Role Norms ^b^	2.68 (0.98)	2.80 (0.97)	2.59 (1.01)
Attitudes Toward Women ^c^	3.55 (0.38)	3.46 (0.50)	3.63 (0.23)
Psychological Distress ^d^	2.01 (0.68)	2.01 (0.69)	2.01 (0.69)
Mental Health Literacy ^a^	3.73 (0.90)	3.91 (0.82)	3.61 (0.96)
Self-Stigma ^a^	2.51 (0.63)	2.49 (0.60)	2.53 (0.66)

^a^ *Scale ranged from Strongly disagree (1) to Strongly agree (5)*

^b^ *Scale ranged from Strongly disagree (1) to Strongly agree (7)*

^c^ *Strongly agree (1) to Strongly disagree (4),*

^d^ *None of the time (1) to All of the time (5)*

In relation to the other variables of interest, scores on male role norms were less than 3 on a scale of 1–7 indicating that participants from both cricket and football clubs expressed a relatively egalitarian attitude towards women. Psychological distress and self-stigma for seeking help were relatively low on a scale of 1–5, and overall mental health literacy confidence was moderate (close to 4 on a scale of 1–5) indicating participants were relatively confident they could access resources (e.g., GP, internet, friends) to seek information about mental illness.

### Relationships between variables

A series of two-tailed correlations were run to examine the relationships between variables. From Table 2, it can be seen that there were four significant relationships. Interestingly, greater psychological distress was significantly associated with lower family support, and was related (but not significantly) to lower youth mental health literacy confidence (*p* = 0.058). Lower youth mental health literacy confidence was however, significantly associated with higher self-stigma against help-seeking. In relation to attitudes towards women, there was a significant negative relationship with male norms, and a significant positive relationship with club support, indicating that those that have a more egalitarian view were also more likely to receive club support, and were less likely to hold a traditional view of men’s roles in society. Finally,

**Table 2:** Correlation coefficients for the relationships between variables

Variable	*n*	1	2	3	4	5	6	7
1.Club Support	34	–-						
2.Family Support	34	.154	–-					
3.Male Norms	34	-.227	-.222	–-				
4.Attitudes to Women	33	.438^*^	.109	-.563^**^	–-			
5.Psychological Distress	34	.076	-.593^**^	.080	-.039	–-		
6.Mental Health Literacy	33	-.024	.282	-.213	.191	-.333	–-	
7.Self-Stigma	33	.232	-.232	.293	-.248	.282	-.467^**^	–-
8.Age	34	-.015	-.024	-.185	.172	-.070	.307	-.341

^*^ Correlation is significant at the 0.05 level (2-tailed)

^**^ Correlation is significant at the 0.01 level (2-tailed)

### Phase two

In the second phase of the project, young men and parents were interviewed to examine the role sport plays in young men’s lives and sport’s influence on the mental wellbeing of young men in contemporary society.

### Participants

Interviewees were recruited through the online survey in Phase One of the research project. In the survey, participants were asked to indicate interest in sharing their sporting club experiences by leaving their contact details for follow-up by a researcher. Selection criteria were based on the participant group and their availability, ensuring the views and experiences of the whole club—young people (12–17 years), parents/guardians, and club stakeholders—were voiced.

Ten individuals expressed interest in participating in an interview, and all were contacted by the researcher, noting that one phone number was no longer connected so could not be contacted. Five people were interviewed over the phone from 22 June to 7 July 2021. The interviewees ranged in age from 18 to 58 years (*M* = 39.20, *SD* = 16.90). Four participants were male and one female. Two of the participants were young male athletes, another two were fathers and one was a mother. In depth qualitative data from 10 participants

was deemed satisfactory for a study such as this, which was reflected in the repetition of themes (ie data saturation) that emerged.

### Data collection

A member of the research team conducted the interviews. The interviews lasted between 43.93 and 98.30 min (*M* = 64.67, *SD* = 23.20), with the interviewer using the first five minutes to build a rapport by asking about the participant’s sporting background. All participants provided informed consent before the commencement of the interview. At completion, all participants were thanked for their time, and youth participants were emailed a $25 gift voucher. The interviews were conducted over the phone and audio-recorded for transcription purposes, and participants were given the opportunity to review and edit their transcripts.

### Qualitative data analysis

A professional transcribing service transcribed the interview audio. The researcher checked the transcripts for accuracy by listening to selected parts of the audio and reading the transcripts simultaneously. The researcher then thematically analysed the transcripts using the method outlined by Braun, Clarke [23]. The first step was familiarisation, which involved reading and re-reading the transcripts to look for ideas and concepts that helped address the research questions. The next step was to code the data and identify emerging themes. The researcher generated codes while systematically reading through the transcripts, then developed a mind map to group the codes into logical themes. A critical friend reviewed these preliminary themes to help the researcher understand and interpret the data and consider alternative interpretations.

The researcher then reviewed the themes in the context of the coded extracts and defined and named the themes: 1) The sporting club as a second family, 2) Shaping young men’s identities, 3) Respectful relationships, 4) Communication and transparency are key.

### Results: Phase two

A range of issues were discussed within the scope of the in-depth interviews with participants involved in this research. Given the nature of the thematic analysis undertaken, there were themes and sub-themes developed. Within the context of this paper, we will report on the major themes to emerge and discuss accordingly. These themes include (i) The sporting club as a second family, (ii) Shaping young men’s identities, (iii) Respectful relationships and (iv) Communication and transparency is key.

#### Theme 1: The sporting club as a second family

While the role of any sporting club is primarily designed to assist in developing physical skills and abilities to play the sport at a competitive level and contribute to the team, these clubs offer much more than merely physical skills and competencies [24]. Sporting clubs, particularly in Australia where this research was conducted, are integral to youth development that can transcend the physical to provide enormous social and cultural capital [25]. Indeed, sporting clubs in marginalised communities such as low socio-economic status (SES) regions and rural and regional areas are often the central point around which the communities revolve [26]. They offer the potential to provide capacities beyond the club's primary focus, namely sporting participation and success, to enable individuals and communities to flourish. Sporting clubs in all regions are key drivers of socialisation as they provide the opportunity for like-minded people with similar interests to a specific location. The physical club site then becomes the place where they can meet and socialise regularly. For a number of the participants, this is akin to a family setting. Quite literally as one of the parents claimed, when asked about the significance of the sporting club in their lives:

The sporting club is, if they spend a fair bit of time there, it almost becomes a second family. And to that end, then you need that – those sort of parental figures. But the good thing is they can chat to them, and then go home, and not have any other contact with them for a week…,

For one of the men involved in cricket, the club allows him to engage with other males irrespective of age or demographic.

Like some of my teammates, I’ve had, like I said, like the old B grade captain. He is 40 years old, and I am 25. Like, he’s a real good mate, like there’s blokes that are 10 years older than me that I have been to their wedding and held their kids and all that sort of thing, and they’re really close mates, and I think it might be because of cricket, because cricket is such a shit game without those teammates to kind of lean on to get you through those tough times.

The same participant went on to identify the significant component as to why he is involved in the sport is based on friendships and being with mates, claiming:

That is part of the bigger reason for us to all play cricket is that camaraderie afterwards, and that team man ship and just hanging out with your mates.

The significance of the club in the overall structure and function of these young men’s lives is not lost on parents. A parent was quick to recognise that his child was in an environment that had the potential to challenge him and yet offer the opportunity for redemption in a safe space. It ultimately provides his child with the ideal place to learn skills and abilities while simultaneously succeeding and failing in a nurturing environment. It was stated:

He definitely feels safe and I'm lucky I've got kids that speak about their feelings and I've got one, my middle child suffers from anxiety and depression, so he feels safe around his sporting club. He uses it to deal with some of those issues. When I say anxiety, he uses it to sort of address it and because it’s a comfortable environment, he feels like he can push himself or let himself be – try different things and fail because he gets to repeat it again quickly and try it again, rather than have to sit on that moment it didn’t go so well for him for the next week, he gets to get up and go and try again.

Similarly, when another parent was asked how important they thought their child’s sporting clubs was to their life, she claimed that the club was:

Huge, it’s their release away from school and any other pressure. As much as there can be a pressure to – I call it having multiple circles, so multiple circles of friendship. So if one circle’s a bit harder right now you can find some solace in another circle for a moment. And you actually understand that it’s just – I don’t know, there’s a bigger picture.

Indeed, the sporting club offers something else other than the friends and social circle created at school. The social groups created at schools may not be the ideal fit for some young people as they have come together based on the necessity of attending the particular school. The sporting clubs are a little different in that a choice has been made to pursue a sport with other like-minded individuals. The “fit” within a sporting club may be a little better than the fit created at school.

#### Theme 2: Shaping young men’s identities

The respective sports of Australian football and cricket, which are the sports where this research is grounded, are fundamentally and historically masculinised spaces [3]. Importantly, however, both sports have developed significantly over the years and have ideologically moved with the contemporary social and cultural expectations of gender equity. There are now women’s football and cricket teams and competitions throughout Australia as women’s movement into masculinised sports abound [27]. Similarly, the acceptance of these sports as legitimate spaces for women continues to grow. The women’s cricket World Cup final hosted in Australia is a testament to this. Over 80,000 people attended the Melbourne Cricket Ground and received over 700 million video views on various media platforms [28]. However, historical and traditional ideologies are often difficult to displace. Therefore, many young males grow up with a perception that to be involved in such sports provides a degree of ‘masculine capital’ that places them higher on the social hierarchy amongst their peers. These sports are seen as a rite of passage for many young males [3]. Indeed, sports have historically been a site for the social construction of masculinity [3, 4]. So called ‘blood sports’ [3] and sports that promote heavy collision and potential injury have been seen as the epitome of masculinised behaviour. Significantly, back in the early 1900s, parents of boys from high socioeconomic status sent their boys to boarding schools where rugby was central to the making of men [29]. Rugby was seen as representing the traits and capacities of warfare without the potential to kill. Therefore, young males were encouraged to challenge their manhood through this sport [29].

Concerning this research, there were many comments by the parents and key stakeholders, such as coaches and the young males themselves, that implied the sporting club provided the opportunity to experience certain traditional male rites of passage [3] and “become men”. Community sport in Australia offers the opportunity for boys and adolescent males to play against men. When that occurs, there is generally no point at which the opposition plays differently that will accommodate the variance in age. Australian sporting culture based on a “win at all costs” approach underpins this ideology, to the point where parents, coaches and players sanction it through continued application [3]. As one of the key stakeholders claimed regarding his own experience in cricket:

Coming through as a young kid even though as a 14-year-old I was still expected to be a man when I played senior cricket and still expected to act like a man when you were playing senior cricket. It doesn’t matter whether you were 14 or 40 you are still; I was still expected to be a man.

Noteworthy were the sentiments raised about the ability of the sporting club to provide a nurturing place around which young males could develop, particularly in the absence of potential male role models elsewhere in society. It was identified that sporting clubs, with their mix of older and younger males, who generally respect authority such as coaches and adhere to training regimes, are the ideal men to provide mentorship and role modelling. As one young male claimed:

The reason why I'm so passionate about sport, and we’ve got to have a bit more of a care for it moving forward, is that it was good for me as a young kid. Between 11 and 17, I probably had male role models. My Mum and Dad parted ways when I was about 10 or 11, so my Mum, luckily, she felt so comfortable having good people around me, male role models at the cricket club. And so it was one of those things, it was time to sort of give back; kids obviously get an opportunity to start coaching and all that and I'm pretty passionate about the life lessons you learn in sport and teams. And yeah, you understand it yourself really.

The same participant went on to reiterate what he had claimed about the usefulness of sport in his life as an emerging young man when he claimed:

You probably get as much lessons or understanding and learnings about yourself in sport. Set aside from all your social activities, sport can teach you a lot about yourself.

Another young male sporting participant identified a similar perspective where sport was concerned in his life:

You learn more about I reckon life by just talking to blokes after the game than you do necessarily about some coaches and all that sort of stuff.

For one of the senior males among the cohort, it was clear that the club had provided him with a richness that flowed onto his quality of life. Indeed, the opportunities, kinship and sense of belonging impacted him in such a way he felt indebted to the club beyond his playing years.

The cricket club has been a very big influence on who I am as a human, and when I retire, like even when these older blokes retire and whatnot, I will still play because I kind of feel indebted to the club. I kind of feel like I owe them something.

#### Theme 3: Respectful relationships

Being involved in a team sport such as football and cricket means a range of aspects that need to be considered where relationships are concerned. The diverse group of young men in these clubs and which this research is based upon, identified several key areas that require further exploration. The young males had to navigate relationships with peers of the same age, and they also had to engage with older men in the club and their team. Importantly, one of the key relationships they need to understand and regularly deal with is their coach. Indeed, their coach played a pivotal role in developing team cohesion and attempting to create good young men. Having to appreciate the nuances of being demoted from a team was just one challenge for the young men, which could ultimately impact their long-term goal as high-level players. This, according to one of the players, was an important learning component for the young men involved. Having a firm and respectful coach of them and listened to their needs played a big part in their development as young men. As one of the young men stated:

The coach and I actually had about an hour conversation after a game once just on players, and who we reckon is going to play. And his coaching style, and where he needs to improve. And he got all this feedback from me, and I was quite surprised. I was like, “wow, I didn’t know he valued it (my opinion) that much”. But I mean, yeah, it showed that he did. So yeah, you could see how he adapted the next week at the next game.

The same player claimed the reciprocal nature of the feedback and how this positively influenced him.

He’s always been honest with me with my feedback in games. So, I mean it’s sort of a two-way street. And I mean, yeah, it’s sort of put everything on the table. Nothing’s going to hurt anyone’s feelings. It’s going to just make him a better coach, or better players. So, I guess we’ve always had that mutual respect.

And;

You know what he expects from you. And they’re all simple things. They’re always laid out. And I guess I followed them well enough for him to respect me. So, it’s sort of yeah, once you’ve got it going one way, it sort of goes both I reckon.

It is these types of relationships that are crucial in the development of young males into respectful men. Significantly, the team's coach cannot be underestimated in terms of the role that he plays concerning the development of these young men.

#### Theme 4: Communication and transparency is key

There are many aspects of mental health that can be positively impacted by communication [30]. Indeed, communication is a key component of a range of crucial elements concerning males and their health more broadly and not solely mental health. However, in the context of this research, with its roots firmly planted in mental health and well-being, the element of honesty and transparency through communication was raised as an important component. By providing ongoing open and honest communication, it was argued that the young males had a greater capacity to understand and interpret the information surrounding issues associated with their sporting performance, which often had implications for team selection. Knowing why an individual is not being promoted over others or being demoted from teams was crucial in playing a part in the young men’s development both as a sportsman and as an individual. One of the parents identified the problematic issue associated with not being open and transparent, which impacted their son and the functioning of their family. Therefore, both a mental health and pragmatic concern manifested into a broader family issue. Regarding team selection, they claimed:

You’re not actually selected until the day before, and often when it comes out on the Friday, it’s a whole different way of living. So, you would plan what you’re doing for the weekend, but you can’t when you play cricket. It comes out – the team is posted usually Friday morning. The kids are usually at school, so there’s no backup system to deal with if you’re dropped a grade or not, or whatever. You’re not in A’s and B’s, it’s Red’s and White’s in cricket, but you know what I mean. So therefore, there’s never – there’s no support system there. So, the kids would have to deal with it at school and then come home that night and then you’re sort of dealing with the fallout of why you’re there.

Not only was there a lack of communication regarding team selection, but there also tended to be broader communication issues surrounding overall individual development. This has numerous implications for development and overall self-esteem. As a parent claimed:

There was a lack of open communication as to why they’re where they’re at. They would go for weeks, sometimes months at a time, where there’s no conversation with coaches about selection.

The claim above is certainly problematic for young males who, for many, are seeking to develop into elite sportspeople. The clubs involved in this research, while community based, are promoted and operated as pathways to the elite national and international system. Some of the men they are teammates in the senior grades are currently, or have been, state and national representatives. This was the case for the cricketers in this research in terms of State and Test cricket. While in the context of Australian football, there are men who have played in the Australian Football League, which is the highest level in the nation. The young males recognise these players and know of their past deeds and accolades in the sporting arena. A number of the young players aspire to be like the elite sportsmen. However, in the absence of regular conversation, some of these young men may struggle to meet the expectations placed upon them from high achieving junior sporting years and a lack of nurturing environment, which can sometimes occur in the transition to the senior sport. As a consequence of this lack of conversation, the potential for young males to drop out of the sport, as one parent identified, is high:

It’s hard to watch so many kids just walk away from the sport and there’s very few that actually go through from juniors to seniors. And they (the club) kept saying, “oh, I wonder why”? And I’m like, “well, all your quality kids who have worked hard to get where they’ve got and they often don’t get the entitlement (rewards) that they deserve.” And then there are some kids that are there – because of their parents – I’m not saying they’re all like that, don’t get me wrong. There’s a lot of kids there that should be, but there’s too much of it where it’s not. It’s just not fair. It’s as simple as that. When you watch what’s going on and you think, “gee, you’ve lost some really quality kids”. Had you actually had the conversation with them? I mean, let’s face it, mental health is a tough thing at that age group and there’s so much going on for them that even just to have a friendly conversation with them by the coach or by – not just the coach. I mean, there’s lots of people that help out at trainings. Those people that help out at trainings could just say something like, “you’re actually doing really well, maybe you could improve on this”. I’m all for telling your kid where they can improve and whatever and the kids want to know. But just communication, it’s as simple as that. It’s just open communication about everything that is going on.

While communication in the eyes of parents was viewed through a lens in terms of what the club and coach could provide young males, a young male athlete highlighted a key area of concern facing young males, and young people more broadly, regarding communication. As a 25-year-old male, this participant claimed to feel like an “older statesman” and that he was viewed somewhat differently from the younger, yet highly talented 15- and 16-year-old boys coming through. For example, even the act of reading a newspaper, which he often did, made this 25-year-old feel antiquated in the presence of these young males given that their mode of communication and “life” revolved around a mobile phone. He stated:

It’s just because, I am 25 I feel like I am an old man reading the newspaper. I mean, it’s okay to be reading the paper (while the game is on) but sitting on your phone is not.

It was apparent that this “older” male, despite being young was raised as a child slightly differently in the sense that he did not have a mobile device such as a mobile phone, until his mid to late teens, which is representative of the times. However, young males who were born just 10 years later have been thrust into a life where “smart phones” are the norm and almost an expectation beyond 10 years of age. The thought of reading physical newspaper would not likely be on their minds.

It is clear with the advent of mobile phones and easy access to social media and electronic messaging these young males under the age of 20 years do not engage in the same types of communication as those in their mid 20 s and beyond. This is a source of frustration for some older males particularly when they have been used to engaging with their fellow team mates fact-to-face whereas some of the younger males engage through messaging apps despite being only several meters away. Additionally, this is argued to have implications for a range of other issues including concentration and patience. One athlete in his mid 20 s stated:

We will have blokes that will sit in the changerooms after the game, and they will send Snap Chats to their group Snap Chat, so yes there are some people in the room. Like there are people out of the room but there will be blokes in the room, so they will kind of communicate through that. It’s like you are across the room just yell out to them, and I think, and I also see, like, everything needs to come very quick, come fast, come lucky, you know what I mean. It’s self-gratification when you put up a photo and everyone likes it and all that sort of stuff, but when it comes to sport, I also think that doesn’t help them when it comes to sport. Like cricket is a long game, but you’ve got to concentrate. It goes for eight hours. The concentration at the cricket is hard for an adult. It’s hard for a man that has done it for 20 years. Like, I don’t think the kids have that and they also have like the patience. They like it if it comes quickly. They are not necessarily willing to be patient – okay I’ve made a change. You’ve got to give it two months to see that change as it comes through. So, make the change in week 1 and they hope it’s not going to work and they don’t take the criticism as well, and all that sort of stuff. It’s just that generally it’s easier for someone to have a conversation via text message than it is to have it face to face.

## Discussion

Mental health, and mental health literacy, has increasingly become an important part of contemporary western culture. Historically, mental health issues were seen as more feminised conditions [14] and therefore taking ownership of a mental health issue was a difficult task for men of all ages and even more so for younger males [3]. Sport offers the ideal space in which to challenge masculinised norms around mental health and promote a site in which to enhance mental health literacy [30–34]. This paper highlights the range of issues that young males within a masculinised sporting setting can face through their daily lives. The mixed-methods data collected from male sporting participants aged 15–24 within the masculinised Australian sports of cricket and Australian football and from parents and key stakeholders such as coaches, board and committee members provide depth and breadth concerning these issues.

The quantitative data indicated that the young men in the sample generally agreed that their sporting club provided them with emotional support and they were able to talk about their problems. However, this was even more so the case for family support. Thus while the quantitative findings did not provide strong evidence to suggest the significance of sporting sites as being important places to shape young men’s positive mental health attitudes, they were identified by the players, parents, and key stakeholders, within the rich descriptive qualitative data, as sites around which they could shape young males into “good young men”. In addition, within the quantitative phase, greater club support was significantly associated with more egalitarian views. With the capacity for strategic leadership and mentoring, the clubs could positively influence young males through their sporting programs. Developing respectful relationships was integral to this component, which could shape these young men’s identities. The need for open and ongoing forms of communication with the young males was perceived, particularly by the parents, as a crucial part of what “the club does” to develop these young males and allow them to enjoy and continue on their sporting “journey.”

It is clear from analysing the data that the sporting clubs at which these young males are located can provide important support networks and enhance mental health. The support networks cannot be underestimated in terms of the health providing factors that come with socialisation. Significantly, the notion of communication was identified as a key element within all of this. Communication between the players and the coach was recognised as crucial in staving off issues associated with poor self-esteem and potentially dropping out of the sport. Indeed, the consequences of dropping out of the sport could ultimately mean the diminishing of broader social support networks given the clubs’ ability to create an important space for the young males to engage with one another and interact at a meaningful level.

Concerning mental health, this research indicates that sporting clubs offer the opportunity to engage with young males regularly. The clubs, therefore, can engage regularly and positively influence them on issues of particular significance, including mental health. However, they also can influence a form of masculinities that tend to conform with traditional masculine attitudes and behaviours and do not often espouse health promotion, including mental health, or tolerance to a range of social and cultural issues such as sexualities and gender diversity [35]. Therefore, the opportunity for sporting clubs to challenge stereotypical notions of masculinity could be a crucial area of influence that can impact factors far beyond a singular area of focus relating to men. Tolerance, understanding, acceptance and the promotion of open communication could be key elements of traditional masculinised sporting clubs in the future. While these are not perceived as core components of such clubs, given the strong emphasis on winning [3], they could be, and ultimately should be, developing and maintaining good young men. This will ultimately provide positive benefits to the communities in which they live and society more broadly.

As with any study, there are a number of limitations that need to be considered in the overall assessment of the results and findings. While the study was robust in its methods there were limited numbers of participants across both phases (particular the quantitative phase). Future research should sample a broader range of athletes from a variety of sporting settings. In addition, our focus was only on football and cricket. Future research could usefully examine whether there are differences across sport type in relation to levels of club support and mental health. The cross-sectional nature of the study also precludes any causal conclusions being made. However, the findings do highlight the importance of mental health and wellbeing as a key element of sporting club culture that needs to be further examined. They also highlight the need for traditional masculinised sporting clubs in becoming inviting and nurturing spaces for young males and for future research on the value of traditional and contemporary education initiatives, including virtual reality, social media, and mobile app technologies to support mental health.

## Conclusion

Sport is a significant factor within the social construction of masculinity in males in western culture. Some claim it is a rite of passage for a range of young males throughout their developmental years [3]. We know that many young males pass through a sporting club at some point in their developmental years, whether for a short or extended period. Indeed, the highly masculinised sporting sites associated with traditional, hegemonic masculinities, including Australian football, cricket, rugby league and rugby union, are important locations for potential change. The need to engage sporting clubs to create nurturing spaces while challenging stereotypical masculine norms is going to be crucial in changing young males’ attitudes and behaviours towards a range of health-promoting behaviours, including mental health. Sport can become an important vehicle for change under the appropriate conditions, and creating those conditions is the next challenge.

## Data Availability

N/A.
